# Estimation of Relevant Variables on High-Dimensional Biological Patterns Using Iterated Weighted Kernel Functions

**DOI:** 10.1371/journal.pone.0001806

**Published:** 2008-03-26

**Authors:** Sergio Rojas-Galeano, Emily Hsieh, Dan Agranoff, Sanjeev Krishna, Delmiro Fernandez-Reyes

**Affiliations:** 1 Division of Parasitology, National Institute for Medical Research, London, United Kingdom; 2 Department of Computer Science, University College London, London, United Kingdom; 3 Department of Infectious Diseases and Immunity, Faculty of Medicine, Imperial College London, London, United Kingdom; 4 Division of Cellular and Molecular Medicine, Centre for Infection, St George's University of London, London, United Kingdom; IBM Thomas J. Watson Research Center, United States of America

## Abstract

**Background:**

The analysis of complex proteomic and genomic profiles involves the identification of significant markers within a set of hundreds or even thousands of variables that represent a high-dimensional problem space. The occurrence of noise, redundancy or combinatorial interactions in the profile makes the selection of relevant variables harder.

**Methodology/Principal Findings:**

Here we propose a method to select variables based on estimated relevance to hidden patterns. Our method combines a weighted-kernel discriminant with an iterative stochastic probability estimation algorithm to discover the relevance distribution over the set of variables. We verified the ability of our method to select predefined relevant variables in synthetic proteome-like data and then assessed its performance on biological high-dimensional problems. Experiments were run on serum proteomic datasets of infectious diseases. The resulting variable subsets achieved classification accuracies of 99% on Human African Trypanosomiasis, 91% on Tuberculosis, and 91% on Malaria serum proteomic profiles with fewer than 20% of variables selected. Our method scaled-up to dimensionalities of much higher orders of magnitude as shown with gene expression microarray datasets in which we obtained classification accuracies close to 90% with fewer than 1% of the total number of variables.

**Conclusions:**

Our method consistently found relevant variables attaining high classification accuracies across synthetic and biological datasets. Notably, it yielded very compact subsets compared to the original number of variables, which should simplify downstream biological experimentation.

## Introduction

High-throughput genomic and proteomic screening of biological samples produces large data arrays [1]–[3] characterizing instances of two different conditions in a very high dimensional space; that is, the space consisting of a vast number of observations or variables that are acquired for each sample. This is the case for mass spectrometry profiles of complex protein mixtures with hundreds of measures of mass-to-charge ratios for polypeptide chains detected in samples such as serum, or genomic microarray studies profiling tens of thousands of genes expressed in tissue samples. The computational analysis of these biological datasets involves the discovery of informative patterns between sample instances and the identification of the specific biomarkers of disease. These analyses facilitate the design of new diagnostic tests or can be used to focus further biological research on specific drug or vaccine candidate molecules. Intuitively, such patterns should not span the entire spectrum of observations but ought to be encoded in a few relevant variables, with the remainder representing noise. The search for such a subset of relevant variables would imply an exhaustive test of all possible combinations, a task that even for the dimensionality of serum proteomic datasets would prove unfeasible. The computational complexity of such searches increases exponentially with the number of variables; it is known as a NP-complete problem and hence computationally intractable [4], [5]. Consequentially heuristic methods with the aim of selecting an approximate-best variable subset must be considered.

There are two approaches to variable selection: filter and wrapper methods [6]. Filter methods rank the complete set of variables with a given criterion, independently from the applied classifier. They have been widely-used in the analysis of proteomic signatures of diseases such as prostate cancer, sleeping sickness and tuberculosis [7]–[9]. Several variants which have also been applied to genomic cancer datasets include lists of permutations of significant variables that are filtered by genetic algorithms (GA) coupled with support vector machines (SVMs) [10]–[13]. Wrapper methodologies on the other hand, implicitly use the classifier to evaluate variables according to their contribution to its predictive power. Although variable selection using wrapper strategies may incur extra computational costs, this is compensated by the ability to explore complex associations between variables detected within the intrinsic patterns incorporated in the discrimination rules. Recursive feature elimination (RFE) uses SVM functions to iteratively rank and discard relevant variables via a greedy search and has been applied to cancer microarray datasets [14]–[18]. The main drawback of this approach lies in the greedy strategy that may disrupt relationships between variables discarded in different stages of the algorithm, leading to sub-optimal selected subsets. To sidestep this difficulty, an alternative approach combines weighted kernels with SVMs [19]–[22]; this approach assigns a weight to each variable to indicate its relevance. In [19] the weight vector is computed using a gradient-descent formulation, which uses bounds on the expected generalization error of the SVM. However, the applicability of this method is restricted by assumptions requiring the kernel and objective functions to be continuous and differentiable, as well as the dataset being separable. In a previous work [22] we proposed to adapt the weighted-kernel SVM using a GA instead of the gradient descent algorithm to improve model selection on weighted radial basis kernels rather than to select variables. In a similar direction, a recent technique using evolutionary strategies to adjust both scaling and orientation of generalized Gaussian kernels in SVMs has been reported [23]; the evolved matrices, however, must be constrained to meet the requirements of proper kernels and, similarly, the aim is to improve the performance of classification instead of selecting variables.

The wrapper method we describe in this paper focuses on estimating a relevance distribution encoded by the weight vector; such a distribution becomes instrumental in the selection of significant variables. For this end, the *weighted Kernel-based Iterative Estimation of Relevance Algorithm* (wKIERA) combines a stochastic-search estimation of distribution algorithm with a kernel pattern-recognition method. The motivation behind using a stochastic estimation of distribution algorithm [24] is three-fold: (i) the ability to derive the parameters of the weighted kernel directly from the resulting relevance distribution; (ii) its capability of avoiding premature poor convergence on optimization of multiple-minima cost functions; and (iii) the low memory-space requirements arising from its compact representation, which is advantageous in the case of dimensionalities of hundreds or thousands of variables. The advantage of employing kernel-based classification is its ability to handle nonlinear decision surfaces in data generated from high-throughput experiments while still adhering to the simplicities of linear classifiers. We reduced the computational cost of the iterative estimation algorithm by using a kernel perceptron [25] as an alternative to SVM, since it provides fast operation with guarantees on upper bounds of misclassification errors. Consequently, wKIERA combines the exploration-exploitation trade-off exhibited by probabilistic model-building stochastic search algorithms for combinatorics [26] with robustness to nonlinear concepts in high-dimensional spaces provided by kernel-based pattern analysis [27]. Our framework successfully selects relevant variables in high-dimensional proteomic and genomic profiles of complex biological processes.

## Results

**Figure 1:**
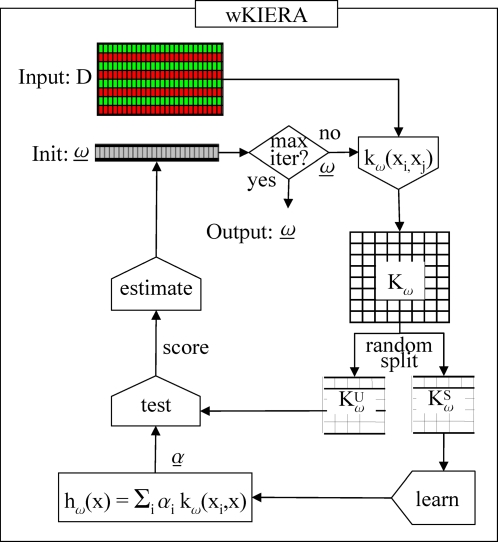
High level flow chart of the *wKIERA* Algorithm.

**Figure 2:**
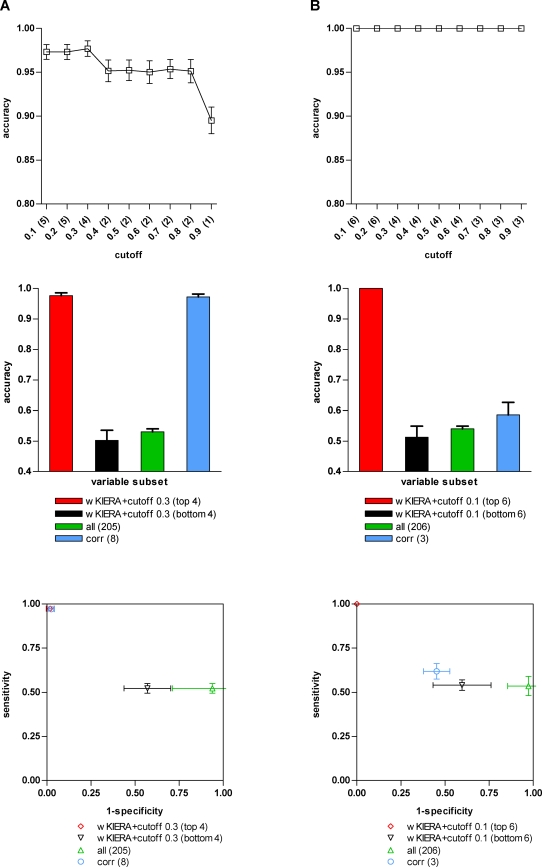
Performance of variable subsets on simulated datasets. A) LOI dataset (wKIERA settings: *poolsize* = 10, *maxiter* = 400, *rep* = 2000, wkRBF ρ = 0.1); B) NLG dataset (*poolsize* = 10, *maxiter* = 400, *rep* = 2000, wkPoly d = 2). Top: Average SVM accuracy on 100 randomly train/test splits using subsets of variables obtained by thresholding the estimated factors of a weighted kernel with the corresponding cutoff on horizontal axis. Resulting subset size (number of variables) is shown in brackets. Middle: Comparison of classification accuracy of SVM trained using variables selected by best-wKIERA-ranked (red); worst-wKIERA-ranked (black); rank correlation coefficients (blue) and using all variables (green). Results are averaged over 100 randomly training/test splits. Bottom: ROC-space analysis of the SVM classifiers shown in the mid plot.

**Figure 3:**
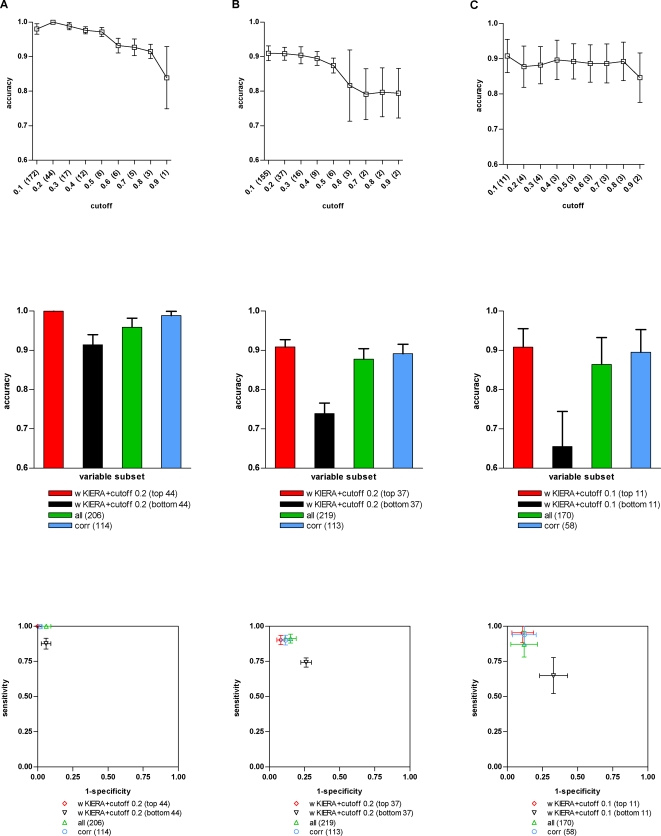
Performance of variable subsets on proteomic datasets. A) HAT dataset (wKIERA settings: *poolsize* = 10, *maxiter* = 400, *rep* = 2000, wkRBF ρ = 0.01); B) TB dataset (*poolsize* = 10, *maxiter* = 400, *rep* = 2000, wkRBF ρ = 1). C) MALARIA dataset (*poolsize* = 10, *maxiter* = 400, *rep* = 2000, wkRBF ρ = 1). Top, Middle and Bottom: See legend on Figure 2.

**Figure 4:**
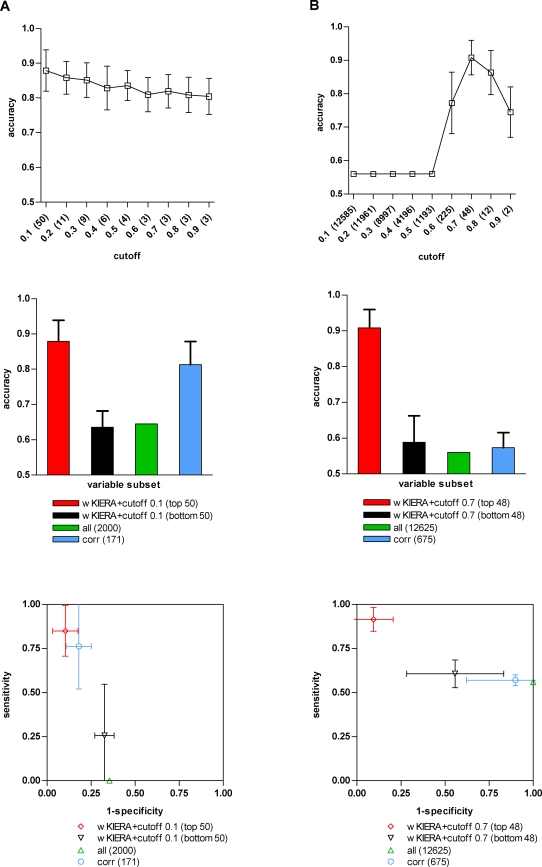
Performance of variable subsets on gene expression microarray datasets. A) COLON CANCER dataset (wKIERA settings: *poolsize* = 100, *maxiter* = 1000, *rep* = 1000, wkRBF ρ = 0.1); B) GLIAL CANCER dataset (*poolsize* = 100, *maxiter* = 1000, *rep* = 1000, wkRBF ρ = 1×10^−5^). Top, Middle and Bottom: See legend on Figure 2.

We performed experiments with wKIERA (Fig. 1) on a variety of synthetic and biological datasets (Table 1). First, wKIERA was run *N* times with different random training/test splits, obtaining an average relevance vector 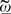. This vector was then scaled to the interval [0, 1] and its components were sorted in descending order with highest values representing relevant variables. We selected relevant variables by defining a cutoff threshold on 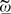. We then used SVMs to evaluate the performance of selected variables in 100 classification experiments using random training/test splits of the dataset. We visualized the classification performance of the subsets of variables obtained by applying a threshold with a step size of 0.1 to the wKIERA relevance vector 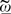 (Figs. 2, 3 and 4 top). We then compared the subset of best performing variables from the threshold plot, with the least relevant ranked variables by wKIERA, as well as with the complete set of original variables and with those rated as relevant according to rank correlation coefficients (Figs. 2, 3 and 4 middle). The performance in ROC space for the same subsets of variables is also shown (Figures 2, 3 and 4 bottom).

**Table 1:** Description of simulated and biological datasets used in this study.

Dataset	Size	D	R	Description	Ref
Linear with redundant variables (LR)	200	206	6	Occurrence of each condition is equiprobable. Six relevant variables are drawn as {*yN*(1,1), *yN*(2,1), *yN*(3,1), *N*(0,1), *N*(0,1), *N*(0,1)} with prob. *p*, otherwise from {*N*(0,1), *N*(0,1), *N*(0,1), *yN*(1,1), *yN*(2,1), *yN*(3,1)}. The remainder variables are drawn from *N*(0,20) The first six variables have redundancy. See ref. for details.	[19]
Linear with outlier variables (LOV)	200	205	5	Occurrence of each condition is equiprobable. Five relevant variables are drawn from 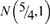 for a positive sample and 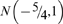 for a negative. The rest are drawn from *N*(0,1). Outliers in variables are induced by selecting 5% of values on relevant variables and re-drawn them from either 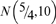 or 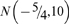 depending on the label. See ref. for details.	[15]
Linear with outlier instances (LOI)	200	205	5	Same method as LOV but this time “instance” outliers are artificially induced by picking 5% of the total samples and re-drawn them from the same distribution with an 10-fold augmented standard deviation. See ref. for details.	[15]
Linear hyperplane (LH)	200	205	5	Five relevant variables are drawn from normal distribution, *N*(0,1). A random normally-distributed hypothesis vector **h̅** is used to label positive samples when x*h̅ **′≥0** and negative otherwise. The remainder variables are drawn from *N*(0,20).	N/A
Nonlinear Gaussian (NLG)	200	206	6	Occurrence of each condition is equiprobable. Negative samples are drawn from multivariate *N*({−¾,…,−3}, *I*) or *N*({¾,…,3}, *I*) with equal probability. Positive samples are drawn from multivariate *N*({3,…,−3}, *I*) or *N*({−3,…,3}, *I*) with equal probability. The rest of variables are noise sampled from *N*(0,20). Relevant variables have redundancy. See ref. for details.	[19]
Nonlinear checkers (NLC)	500	202	2	All variables are drawn uniform randomly from the interval [0,1]. Condition label is determined as the logical exclusive-OR between the first 2 variables, *y* = *XOR*(*x*_1_,*x*_2_). The resulting 2-dimensional subspace of relevant variables resembles a 2×2 checkerboard. The rest of variables are noise sampled from *N*(0,20).See ref. for details.	[13]
Human African Trypanosomiasis (HAT)	231	206	?	SELDI-ToF Proteomic dataset of 85 serum samples from patients affected with Human African Trypanosomiasis (sleeping sickness) plus 146 control serum samples. See ref. for full details on demographics and data gathering.	[9]
Tuberculosis (TB)	349	219	?	SELDI-ToF Proteomic dataset consisting of 179 serum samples from patients affected with active Tuberculosis plus 170 control serum samples. See ref. for full details on demographics and data gathering.	[7]
Malaria	170	56	?	SELDI-ToF Proteomic dataset consisting of 28 serum samples from patients affected with Malaria plus 28 control serum samples. To be published elsewhere.	N/A
Colon cancer	66	2000	?	Publicly available gene expression microarray consisting of 40 tumor and 22 normal colon tissue samples.	[29]
Glial cancer	50	12625	?	Publicly available gene expression microarray consisting of 28 samples of glioblastomas and 22 samples of anaplastic oligodendrogliomas. See ref. for further details.	[30]

D = dimension, R = number of relevant variables.

To assess the framework reliability we designed experiments using linear and non-linear simulated proteomic-like datasets with predefined sets of relevant variables. For all of the synthetic datasets wKIERA selected the correct relevant variables among the first top-ranked components of 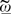 except for the LH dataset where one irrelevant variable was ranked before another relevant (Table 2). Figure 2 shows the classification performance of two representative proteomic-like artificial datasets: one with outlier instances (LOI) and the other sampled from a mixture of Gaussians (NLG). On the LOI dataset, the performance of wKIERA is comparable to that of the rank correlation coefficients but with a smaller set of relevant variables (Fig. 2A middle). The accuracy obtained with the worst-wKIERA-ranked variables is close to random classification as expected and shows that the best-ranked variables were not selected by chance. Moreover, classification using all variables is poor because excessive noise is introduced by the non-relevant variables (Fig. 2A middle). Similarly, in the NLG dataset, classification with selected variables by wKIERA outperformed that of bottom-ranked or all variables (Fig. 2B middle). On this dataset our method clearly outperformed rank correlation coefficients and indeed it is known that the latter method is sensitive to non-linear labeling functions (Fig. 2B middle). Experimental results for the other synthetic datasets followed similar trends (not shown); in all cases the predefined relevant variables were successfully selected by wKIERA (Table 2).

**Table 2:** Selected variables in synthetic datasets by wKIERA (*poolsize* = 10, *maxiter* = 400).

Dataset	10-top-ranked variable index										Matched/true relevant	Kernel settings
LR	***3***	***6***	***2***	***5***	***1***	***4***	45	116	76	191	6/6	wkRBF (ρ = 0.1)
LOV	***2***	***4***	***1***	***3***	***5***	28	53	93	75	7	5/5	wkRBF (ρ = 0.1)
LOI	***4***	***3***	***5***	***2***	***1***	87	132	54	20	142	5/5	wkRBF (ρ = 0.1)
LH	***5***	***3***	***1***	***4***	162	***2***	169	27	191	85	5/5	wkPoly (d = 1)
NLG	***3***	***4***	***1***	***2***	***5***	***6***	141	73	170	78	6/6	wkPoly (d = 2)
NLC	***2***	***1***	178	64	150	162	84	101	3	27	2/2	wkPoly (d = 2)

Type of kernel used in each dataset, weighted RBF kernel (wkRBF) or weighted Polynomial kernel (wkPoly), is showed in rightmost column. Numbers in ***bold-italic*** represent true relevant variables.

We assessed the performance of wKIERA on real data using a panel of high-dimensional biological patterns. We focused our experiments on proprietary proteomic datasets of infectious diseases such as Human African Trypanosomiasis (HAT) [9], Tuberculosis (TB) [7] and Malaria (Table 1). On the HAT dataset, classifiers trained with variables selected by wKIERA achieved an accuracy of 99% with comparable performance to using those selected by rank correlation coefficients (Fig. 3A). However, the number of variables selected by wKIERA was much smaller (21% of the total (44) compared to 55% (114)). Interestingly, classifiers trained with all variables or the worst-wKIERA-ranked subset of variables showed accuracies above 90%, which indicates that discrimination patterns are widely distributed across all variables in this dataset. Analyses of the TB dataset show that wKIERA selected variables yielding an accuracy of 91% while for those selected with rank correlation coefficients the accuracy was 89% and using all variables 87% (Fig. 3B). As on the previous dataset the wKIERA subset was the smallest (17% of total size (37)) compared to 52% (113) and 100% (219). A 74% accuracy obtained by the worst-wKIERA-ranked may indicate the occurrence of noise in this dataset. Lastly, results for the Malaria dataset were wKIERA: 91%, rank correlation coefficients: 89%, and all-variables: 88% (Fig. 3C). Consistently, the subset obtained with wKIERA is much smaller (11 compared to 58 from a total of 170 variables). Once more, the 65% obtained with the worst-wKIERA-ranked may also suggest the presence of noise in this dataset.

In order to assess the scalability of our method to higher numbers of variables, we subsequently conducted experiments on publicly available microarray datasets (Table 1) where dimensionality was increased between two and three orders of magnitude compared to the proteomic datasets described above. On the COLON CANCER dataset, the wKIERA subset of variables achieved 88% accuracy with only 2.5% (50) of the total variables, whereas rank correlation coefficients achieved 82% with a size of 8.5% (171) (Fig. 4A). On the other hand, in the GLIAL CANCER dataset the wKIERA subset attained 91% accuracy, outperforming all the other subsets of variables that achieved accuracies below 60% (Fig. 4B). Again, the small number of variables selected by wKIERA (just 0.4% or 48 out of 12626) is noteworthy. The poor performance obtained with rank correlation coefficients indicates that labeled-correlated variables are insufficient to solve the possibly non-linear separation surfaces contained in this dataset.

In summary, our wKIERA method consistently found relevant variables attaining high classification accuracies in synthetic and biological datasets, and yielded subsets that were very compact compared to the original number of variables. This is highly desirable for the feasibility of downstream biological experimentation. The method reliably scaled-up to dimensionalities of much higher orders of magnitude even when few instances were available, as shown with the cancer microarray datasets.

## Discussion

We propose an iterative framework for weighted kernel-based relevance estimation for high dimensional biological patterns. Variable relevance estimation assuming variable independence was achieved using a kernel perceptron classifier coupled with a probabilistic-model-building stochastic optimizer. We have shown the viability of such a configuration in controlled synthetic experiments. In a set of experiments involving proteomic profiles for infectious diseases our method found sets of significant protein clusters that achieved high classification accuracies but which were three times smaller than sets derived using classic correlation coefficients. The dimensionality of the overall datasets varied between 170 and 219. We also tried our method in problems with much larger dimensionalities such as cancer expression microarrays with 2000 and 12625 genes where only a handful of instances are available. The method scaled-up remarkably well in these situations, revealing significant patterns.

Weighted polynomial or RBF data-pattern kernel representations can be used within the wKIERA framework. Use of weighted RBF kernels was preferred for biological datasets because they are considered to be polynomial kernels of infinite degree [27]. For synthetic datasets such as LH, NLG and NLC we experimented with polynomial weighted kernels in accordance with previous studies in the literature where the non-weighted versions were used [13], [15], [28].

The wKIERA framework modularity admits different configurations where faster online learning algorithms and more complex probabilistic-based search models can be used. This might allow us to analyze complex patterns of composite variable interactions and multivariate dependencies. We are currently investigating new mistake-driven algorithms with better generalization performance than the kernel perceptron but still showing fast execution. We are also considering refining the estimation of distribution algorithm by using probabilistic graphical models to represent higher-degree, nonlinear, conditional, or even time dependencies between variables. This research path may further improve the ability of our method to find informative pattern distributions that are likely to emerge given the dynamic nature of protein interactions.

## Materials and Methods

### Datasets

Proteome-like synthetic datasets were designed in order to perform controlled experiments using dimensionalities of two hundred variables, from which two to six were relevant. We encoded linear and non-linear labeling functions into the relevant variables. A few hundred samples were included, resulting in square-shaped data matrices. Sampling and labeling mechanisms are described in Table 1. We generated four linear datasets: LR, where some relevant variables can be discarded as redundant without disturbing classification accuracy; LOV, where noise was introduced to particular loci in randomly selected instances simulating artifacts generated during array processing; LOI, where noise was imposed on all variables in randomly selected instances, simulating inaccurate collection of samples; and LH, where a predefined linear discriminant for relevant variables was used to label the instances. In addition, two nonlinear datasets were generated: NLG, where clusters of mixtures of Gaussians were generated for each class, and NLC, where the clusters follow a tighter checkers-patterned distribution. The last two datasets also included redundancy.

Experiments were also conducted on real biological datasets. We tested proprietary proteomic profiles of infectious diseases (HAT [9], TB [7] and MALARIA [Unpublished]). These high dimensional datasets are almost square, i.e. the number of variables and instances are similar (Table 1). We also used two publicly available gene expression microarray datasets COLON CANCER [29] and GLIAL CANCER [30]). These datasets have a much higher dimensionality (2000 and 12625 respectively) and fewer instances (66 and 50 respectively). Compared to the proteomic datasets, the latter two datasets are rectangular in shape posing a more challenging obstacle to variable selection because of the curse of dimensionality phenomenon, i.e. shortage of sufficient instances to correctly sample high dimensional spaces.

### Notation

We denote *D* = {(x_1_,*y*_1_),…,(x *_m_*,*y_m_*)} a collection of *m* instance/label pairs where each instance x *_i_* = (*x_i_*_1_,*x_i_*_2_…,*x_in_*) consists of *n* observations representing one sample in an *n*-dimensional space, *y_i_*∈{1, −1} specifying its binary class label, and 1≤*i*≤*m*. The coordinates of such a space are related to variables; each one associated with a factor ω ˆ*_i_*∈{0,1} to indicate its relevance. The vector ω approximates these factors using continuous weights ω*_i_*∈[0,1]. The set of instance indexes is denoted by *J* = {1,2,…,*m*}. Instances are randomly split into a training subset *S* and a test subset *U* to be used by a learning classifier. The kernel matrix of all instances in *D* is denoted by *K*, the kernel matrix of training instances by *K_S_* and the kernel matrix of training versus test instances by *K_U_*. The class labels for training and test sets are denoted by *y_S_* and *y_U_* respectively. A candidate weight vector that approximates the optimal ω is termed *w*. The collection of all such vectors *w* is denoted *W* while the collection of vectors *w* with best classification performance is *B*.

### weighted Kernel-based Iterative Estimation of Relevance Algorithm (wKIERA)

A high level depiction of wKIERA is shown in Fig. 1. The method iteratively optimizes the parameters (ω,α) of Eq. (13) by executing the components marked as *learn* and *estimate*. We used a kernel perceptron as a supervised learner [25] and an estimation of distribution algorithm for the *estimate* component [24]. However, the modular design of the wKIERA allows plugging of any linear-threshold kernel classifier and any stochastic optimization algorithm into these components.

The stochastic optimization module for estimation of ω was designed with a probabilistic model-building strategy known as estimation of distribution algorithm [24] and is summarized in Table 3. Inputs are a dataset *D*, the number of candidate weight vectors *w* (*poolsize*), the maximum number of iterations (*maxiter*), and the parameters of a base kernel. Depending on the kernel type, this can be the degree of a polynomial kernel *d* or the width of a RBF kernel ρ. This base kernel will be transformed to a weighted version using every candidate weight vector *w*.

**Table 3:** Weighted Kernel-based Iterative Estimation of Relevance Algorithm (wKIERA).

Algorithm wKIERA
Inputs
Dataset: D = {(**x**_J_,*y*_J_)}, J = {1,…,m}; Base kernel: kerbase;
Pool size: poolsize; Max. iterations: maxiter;
Output
best**w**
Algorithm
n = dim(**x**_1_);
W = rand_matrix_01 (poolsize,n)
repeat for (t = 1, top = 0; t<maxiter; t++)
[S,U] = random_split (J,n/2)
repeat for each row **w** in W
K = compute_wkernel (**w**,**x**_J_,kerbase)
h = train_kperceptron(K_S_,*y*_S_)
score**_w_** = 0.99*test_kperceptron (h,K_U_,*y*_U_)+0.01*len(**w**)
if(score**_w_**>top)
top = score**_w_**; best**w** = **w**;
end_if
end_repeat
B = select_half_best (W,score_i = 1:poolsize_);
μ = mean(B); σ = std_dev(B);
[δ,ξ] = skewness_schedule (t,top);
W^new^ = μ+((σ+ξ)*rand_matrix_skewed_01 (poolsize,n,δ))
W^new^_1_ = best**w**; W = W^new^
end_repeat

First, the pool of weight candidates *W* is uniformly randomly initialized and the main loop (Table 3) is executed *maxiter* number of iterations. The variables *top* and *bestw* are used to trace the candidate with best score across all iterations. On each iteration the set of instance indexes *J* = {1,2,…,*m*} is split into two subsets of randomly permuted indexes, *S* and *U*. Then a weighted kernel matrix *K* is computed using the corresponding weight vector *w*, the input vectors **x** *_J_* and the base kernel. The kernel matrix *K_S_* is fed into a kernel perceptron to learn a discriminant function *h* that classifies the examples in *S* within a supervised learning framework using the corresponding labels *y_S_*. The fitness of the candidate weight vector *w* is then evaluated with the multi-objective scoring function of Eq. (1) which depends on classification accuracy in the test set using *K_U_* and *y_U_*, and a measure of its length. A matrix *B* is then created with half the best-scoring weight vectors from *W*. The matrix *B* is now used to estimate a uniformly and independently multivariate Gaussian distribution by computing the mean and standard deviation vectors μ and σ. Two additional parameters for noise δ and skewness ξ are set using a predefined schedule of the current iteration number and the *top* score. At this point a new pool of weight vector candidates *W* is generated using the estimated probability distribution with added perturbations. A skewed multivariate normally distributed matrix *W^new^*∼*N*_δ_(μ,σ+ξ) is used for this purpose. Negative values generated by this distribution are set to zero since only positive values are valid weights ω*_k_* in Eq. (10) and Eq. (11). Finally, the best candidate *bestw* is carried over to the next iteration by assigning it to the first slot of the new pool *W* (as suggested in [31]). These steps are repeated a maximum number of iterations or until the algorithm halts for a maximum period of consecutive iterations. At the end of the loop the best candidate *bestw* containing the estimated vector of weights ω is returned.

The [δ,ξ]-schedule was defined according to the best parameters found in preliminary experiments. The amount of noise δ added by the random number generator was initialized in 0.2 and linearly declines to zero by the final iteration. This is intended to encourage a broader exploration of the search space at the beginning stages of the algorithm while further exploitation of the feasible subspace is performed in the later stages. On the other hand, the skewness of the distribution ξ is set to zero up to the point where *top* score achieves a safety-net value of 0.9 when it starts to decrease towards a value of −1. When this happens, the random number generator becomes biased to produce negative weight values which in turn will be set to zero. This is meant to promote downscaling of irrelevant variables in classifiers obtaining high classification scores. A safety-net value of 0.9 will ensure that classifiers with less than 90% accuracy are penalized.

### Scoring function

The score function guides the search of the wKIERA algorithm. It is defined as a multi-objective function made of an estimate of the accuracy of a weighted kernel classifier and a measure of the size of the weight vector:
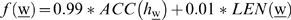
(1)


The first term in Eq. (1), corresponding to the accuracy of a classifier, computes the proportion of correctly classified examples in an unseen test set. Classifiers with higher rates of accuracy get values close to 1. The second term in Eq. (1) is intended to solve ties between candidates with the same accuracy, in which case those with lower scale factors are preferred. For this purpose the average of **w** is used to calculate *LEN*(**w**) = 1−*AVG*(**w**); thus candidates comprising plenty of null weights get length values approaching to 1. We weight the first term of the multi-objective function with 0.99 as classification accuracy should be the dominant criterion of the search.

We consider other measures of classification performance, including sensitivity (SE) and specificity (SP) of a classifier. They are defined in Eq. (2) and Eq. (3), where TP and TN denote the number of positive and negative correctly classified cases, and FP and FN denote the positive and negative misclassified cases. The accuracy, then, can be computed as Eq. (4). We used TP and TN to plot classifiers in a receiver operator characteristic (ROC) space where the performance (positive diagnostic likelihood ratio) of a classifier is expressed by its true positive rate (TPR = SE) and false positive rate (FPR = 1−SP).
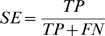
(2)

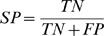
(3)

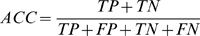
(4)


### Linear-Threshold Discriminants

A linear-threshold discriminant corresponds to a hyperplane in the space of instances in *D*, that is, an *n*-dimensional plane defining two half-spaces. An instance is hence classified as positive or negative depending on the side of the hyperplane it lies on. A hyperplane is characterized by its normal *n*-dimensional weight vector **w** and a bias term *b* (*b*≠0 refers to a non-centered hyperplane). A linear discriminant function can be specified as a rule to discriminate instances in *D*:
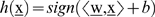
(5)where 〈·,·〉 denotes inner product. By weighting input variables in **x** with ω, the contribution of variables with non-significant factors to the inner product in Eq. (5) is diminished. The linear discriminant therefore becomes:
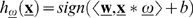
(6)where * denotes element-wise product. The parameters (**w**,*b*) are obtained by solving an optimization problem on the misclassification error incurred by *h* _ω_:
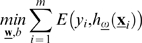
(7)here 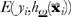 measures the discrepancies between the predicted and the real label on every instance in *D*.

### Weighted kernels

A kernel is a continuous, symmetrical and positive semi-definite function between two vectors in a given Hilbert space *H*. Mercer's theorem [32] states that such a function corresponds to the inner product between images of the input vectors in a transformed feature space (usually of a larger dimensionality). Therefore, when vectors from the input space are mapped to a feature space **x** *_i_*↦φ(**x** *_i_*) using the nonlinear transformation φ(·), their inner products in the feature space becomes 〈φ(**x** *_i_*), φ(**x** *_j_*)〉↦*k*(**x** *_i_*,**x** *_j_*) where *k* : *H*×*H*↦ℜ is a function mapping a pair of points in *H* to the real set ℜ. By means of φ(·), nonlinearities in the input space can be solved with linear discriminants in the feature space if a proper function *k*(·,·) is used. In the present study *H* is defined by ℜ*^n^*.

Two widely-used kernel functions are the Radial Basis Function (RBF) and polynomial kernels defined in Eq. (8) and Eq. (9) respectively:
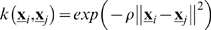
(8)

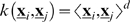
(9)where the parameter ρ>0 is the width of a symmetric radial function similar to a Gaussian bell centered in one of the input patterns and the parameter *d*>0 is the polynomial degree. A weighted version of these kernels assigns a scale factor, 0<ω*_k_*<1, for each input dimension as shown in Eq. (10) and Eq. (11) respectively [19]. In the weighted polynomial kernel the scale factors ω*_k_* adjust the contribution of each variable to the inner product. In the weighted RBF kernel ω*_k_* shape the width of the radial function in every dimension. Null scale factors prevent the corresponding variables affecting the kernel computation, making them irrelevant in practice.
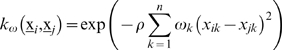
(10)

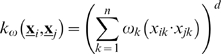
(11)


Any kernel defines a so-called Reproducing Kernel Hilbert Space (RKHS) where an inner product between two arbitrary vectors amounts to the evaluation of the correspondfoing kernel function. In this way a hyperplane in the RKHS can be characterized by replacing inner products with kernel functions and hence the linear discriminant of Eq. (5) becomes [33]:
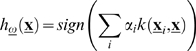
(12)and the weighted version of Eq. (6) corresponding to:
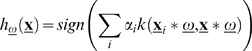
(13)


The expression *k*(**x** *_i_* * ω,**x** *_j_* * ω) in Eq. (13) matches one of the above-defined weighted kernels which we have denoted *k* _ω_(**x** *_i_*,**x**_j_). Note that a kernel matrix *K* _ω_ can be computed off-line for every pair of instances in *D*, i.e. as ⌊*K* _ω_⌋*_ij_* = *k* _ω_(**x** *_i_*,**x** *_j_*).

### Kernel Perceptron

A Perceptron classifier [34], [35] uses a hyperplane to separate examples from a dataset *D* onto different half-spaces corresponding to binary classes. The hyperplane is represented by the parameters (**w**,*b*) of Eq. (5) which are learned by a mistake-driven algorithm conducting incremental updates from a stream of instances. It has been shown [36], [37] that given two separable sets of positive and negative examples in a Hilbert space, the Perceptron algorithm converges to a discriminant hyperplane with a number of mistakes theoretically bounded in terms of the distance of separation between the sets (also know as their *margin*). The linear separability constraint which is certainly difficult to ensure in realistic situations, can be solved by using kernel functions to transform the input space to a higher dimensional RHKS [33]. The resulting Kernel Perceptron algorithm [25], is able to learn a linear discriminant with implicit kernel representations as in Eq. (12). Additional advantages of this algorithm include ease of implementation and fast computation; given its incremental character, the number of updates grows as *O*(*n*) where *n* is the number of examples in *D*.

### Support Vector Machines

The SVM [27], [33], [38] is a kernel machine that learns a hyperplane with the maximal margin of separation between vectors of two distinctive classes in a RKHS. The discrimination function of an SVM is similar to that of the Kernel Perceptron and takes the form showed in Eq. (14),
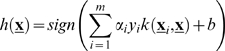
(14)where the coefficients α*_i_* and the bias term *b* are found by solving a constrained quadratic optimization problem aimed to minimize the misclassification rate and the complexity of the classifier while maximizing the margin. Notice that only those patterns whose α*_i_*≠0, participate in the computation of Eq. (14) and hence they are called the support vectors. The motivation for maximizing the margin is rooted in the theory of Structural Risk Minimization [38] and its aim is to maximize the generalization ability of the discriminant by reducing its capacity. In this sense, the SVM learns the optimal separating hyperplane whereas the Kernel Perceptron learns an approximation to that optimum. However the computational complexity of the SVM is quadratic in time since it requires *O*(*n* ^2^) computations to solve the quadratic optimization problem.

### Rank correlation coefficients

The Pearson correlation coefficients are computed using Eq. (15) where *X_k_* represents the random variable corresponding to the *k*-th component of the input instance vectors (*k* = 1,2,…*n*) and *Y* is the random variable representing the class labels.
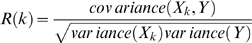
(15)


Since only a finite sample of the input instances is available, the estimate of *R*(*k*) is given by Eq. (16) where *x_ik_* corresponds to the *k*-th variable value of the *i*-th sample and *y_i_* is its class label.
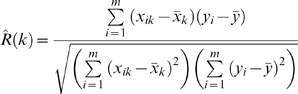
(16)


### Source code

The proposed method was implemented in Matlab 7.0 including scripts for wKIERA, kernel perceptron, scoring and evaluation functions. The source code is available upon request. For evaluation of SVM classifiers we used the SVMLight [39] library with the MEX-SVMLight interface for Matlab [40].
